# Dissecting Oct3/4-Regulated Gene Networks in Embryonic Stem Cells by Expression Profiling

**DOI:** 10.1371/journal.pone.0000026

**Published:** 2006-12-20

**Authors:** Ryo Matoba, Hitoshi Niwa, Shinji Masui, Satoshi Ohtsuka, Mark G. Carter, Alexei A. Sharov, Minoru S.H. Ko

**Affiliations:** 1 Developmental Genomics and Aging Section, Laboratory of Genetics, National Institute on Aging, National Institutes of Health Baltimore, Maryland, United States of America; 2 Laboratory of Pluripotent Cell Studies, RIKEN Center for Developmental Biology Kobe, Japan; Institute of Genetics and Molecular and Cellular Biology, France

## Abstract

POU transcription factor Pou5f1 (Oct3/4) is required to maintain ES cells in an undifferentiated state. Here we show that global expression profiling of Oct3/4-manipulated ES cells delineates the downstream target genes of Oct3/4. Combined with data from genome-wide chromatin-immunoprecipitation (ChIP) assays, this analysis identifies not only primary downstream targets of Oct3/4, but also secondary or tertiary targets. Furthermore, the analysis also reveals that downstream target genes are regulated either positively or negatively by Oct3/4. Identification of a group of genes that show both activation and repression depending on Oct3/4 expression levels provides a possible mechanism for the requirement of appropriate Oct3/4 expression to maintain undifferentiated ES cells. As a proof-of-principle study, one of the downstream genes, *Tcl1*, has been analyzed in detail. We show that Oct3/4 binds to the promoter region of *Tcl1* and activates its transcription. We also show that Tcl1 is involved in the regulation of proliferation, but not differentiation, in ES cells. These findings suggest that the global expression profiling of gene-manipulated ES cells can help to delineate the structure and dynamics of gene regulatory networks.

## Introduction

Mouse embryonic stem (ES) cells, derived from the inner cell mass (ICM) of blastocysts [1], [2], can be maintained indefinitely *in vitro* [3] in an undifferentiated self-renewing state in the presence of Leukemia Inhibitory Factor (LIF) [4], [5] and Bone Morphogenetic Proteins (BMPs) [6]. The cells in culture can also retain their ability to differentiate into all three germ layers both *in vitro* and *in vivo*, and are thus as highly pluripotent as their *in vivo* counterparts, ICM cells. The mechanisms underlying these phenomena have been extensively studied [7]–[11], but it is likely that many of the genes and regulatory mechanisms involved have yet to be identified.

Two transcription factors, *Oct3/4* (*Pou5f1*: POU domain class 5 transcription factor 1, also known as *Oct3* and *Oct4*) [12]–[14], and *Nanog* (variant homeobox protein) [15], [16] are known to play important roles in mouse ES cells. The expression of Oct3/4 is restricted to pluripotent embryonic and germline cells [17]. In blastocysts, Oct3/4 protein is more abundant in the ICM than in trophectoderm cells, but in late blastocysts Oct3/4 protein is even more abundant in primitive endoderm than in the ICM [18]. *In vitro*, the reduction of Oct3/4 expression fosters differentiation of ES cells into trophectoderm, i.e., placental lineage, whereas the overexpression of Oct3/4 promotes differentiation into primitive endoderm and mesoderm [19]. However, Oct3/4 cannot maintain the undifferentiated state of ES cells without LIF. Therefore, Oct3/4 is required but not sufficient for the maintenance of undifferentiated ES cells [19], [20]. In contrast, the recently identified Nanog gene can bypass LIF-pathways and maintain ES cells undifferentiated in the culture media without LIF. However, Oct3/4 is demonstrably required for Nanog function, because forced repression of Oct3/4 will allow ES cells to differentiate independent of Nanog levels [8].

A first step to understand the function of *Oct3/4* is to identify downstream target genes. *Fgf4, Utf1, Spp1/Opn, Fbxo15/Fbx15, Sox2, Pdgfa/PDGFα, Cga/α,βhCG, Ifngr1/τINF*, and *Zfp42/Rex1* have been identified [9], [21]–[27]; and screening by cDNA subtraction methods have also identified *Otx2, Lefty1*, and *Upp1/Upp* as potential downstream target genes [28]. Recently *Dppa5/Esg1* [29], [30] and *Nanog* [31], [32] have been added to the candidate gene list. Some of these target genes are expressed specifically in ES cells as well as in the ICM of blastocysts. However, their functions are largely unknown, and Oct3/4 has not yet been linked to or associated with critical physiological pathways.

Recently, genome-wide surveys of Oct3/4-binding sites have been performed on human ES cells by chromatin-immunoprecipitation (ChIP)-on-chip assays [33] and on mouse ES cells by ChIP-PET assays [34]. In human ES cells 581 genes have been identified as Oct3/4-target genes, whereas 963 genes have been identified in mouse ES cells. These studies have provided a list of genes that are primary downstream targets of Oct3/4, and demonstrated physical associations between transcription factor proteins and promoter sequences *in vitro*. On the other hand, ChIP-on-chip or ChIP-PET assays do not provide information about whether these primary target genes are activated or repressed by Oct3/4 binding. In addition, secondary downstream target genes, which are regulated by the primary target genes, cannot be identified by such studies.

We have manipulated Oct3/4 levels in ES cells and carried out expression profiling by microarray analysis to identify new candidate genes as downstream targets of Oct3/4. We then followed up one of them, Tcl1, as a pilot for systematic analyses, and confirmed that it is indeed transcriptionally regulated by Oct3/4 and involved in the control of ES cell proliferation.

## Results

### Global gene expression changes in Oct3/4-manipulated ES cells

To study overexpression of Oct3/4, we used ZHTc6 ES cells, i.e., *Pou5f1* ^+/−^ ES cells transfected with a Tetracycline (Tet)-regulated *Pou5f1* transgene [19]. In this *in vitro* system, withdrawal of Tet overexpresses Oct3/4 and differentiates ES cells into cells similar to primitive endoderm and mesoderm. For Oct3/4 repression studies, we used ZHBTc4 ES cells, which were made by disrupting the remaining *Pou5f1* allele of ZHTc6 ES cells [19]. Although both *Pou5f1* alleles were disrupted, the ZHBTc4 cells can be maintained as undifferentiated by the continuous induction of an *Oct3/4* transgene in the absence of Tet. Addition of Tet to the culture media represses Oct3/4 and provokes differentiation of the ES cells into cells similar to trophectoderm [19].

**Figure 1:**
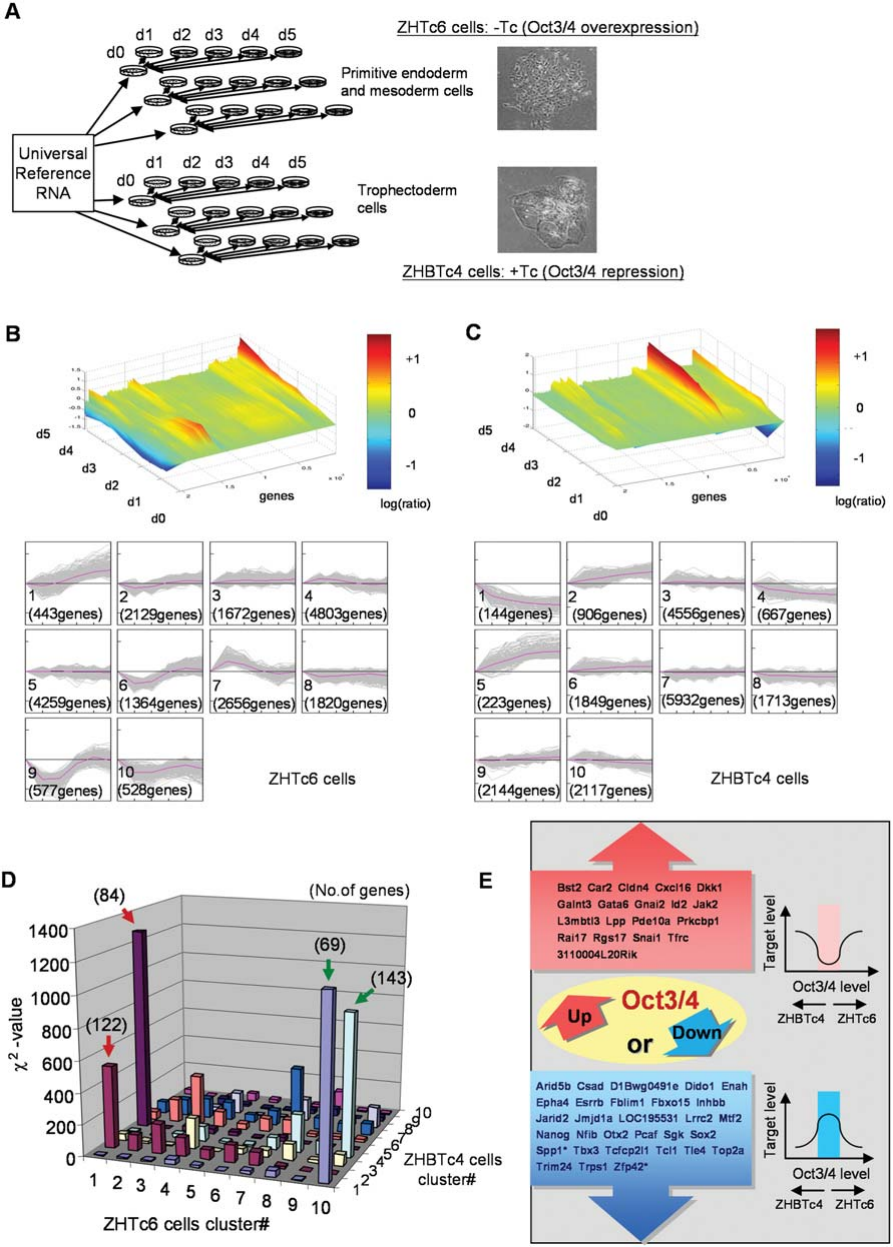
Global gene expression analysis of *Oct3/4* manipulated ES cells. (A) Experimental design of microarray analysis. Oct3/4-overexpressing ES cells differentiate to primitive endoderm and mesoderm, whereas Oct3/4-repressed ES cells differentiate towards Trophectoderm. (B)(C) K-means clusters of gene expression levels analyzed by TIGR MEV tool (http://www.tigr.org) and their landscape representations by MATLAB. (D) Comparison of genes grouped in each cluster of ZHTc6 cells and ZHBTc4 cells. The arrows (red and green) show highly correlated clusters between two cell lines (see Materials and Methods). (E) Diagram summarizing the unique mode of gene expression regulation by Oct3/4. Representative Genes identified as the direct downstream targets of Oct3/4 by the ChIP-on-chip assay [33] or ChIP-PET assay [34] are shown. Spp1 and Zfp42 (marked with asterisk) were not detected as a direct downstream target by either ChIP-on-chip or ChIP-PET assays, but have already been shown as such by more direct experimental methods (see the text).

We carried out expression profiling of the ES cells in triplicate at five time points (every 24 hrs: day 1, 2, 3, 4, and 5) after withdrawal of Tet in ZHTc6 ES cells (for overexpression of Oct3/4) or after addition of Tet in ZHBTc4 ES cells (for repression of Oct3/4) using a 22K 60-mer oligonucleotide microarray [35] (Figure 1A). As expected, in ZHTc6 cells the *Oct3/4* expression level was induced by 1.2-fold at day 2 and 2-fold at day 3 after withdrawal of Tet. In ZHBTc4 cells, the *Oct3/4* expression level immediately fell 5-fold and was maintained from day 1 after adding Tet. These results were consistent with a previous report [19] and were further confirmed by real time Q-PCR (data not shown).

As a first step toward assessing global trends, we identified changes in expression levels of individual genes by examining 20,251 genes that showed consistently-replicated expression levels (Supplemental Table S1). The expression patterns of these genes in ZHTc6 cells and ZHBTc4 cells were analyzed separately using a k-means clustering algorithm and grouped into ten clusters (Figure 1B, C). Upper panels show the expression changes of individual genes in a 3D landscape map, whereas lower panels show the pattern of averaged gene expression changes and the number of genes grouped into each cluster. There were marked differences in the expressions of many genes between ZHTc6 cells and ZHBTc4 cells. In ZHBTc4 cells, genes showed relatively consistent trends over 5 days, whereas in ZHTc6 cells, larger numbers of genes responded rapidly, but were restored to original levels by day 3 (Figure 1B, C). The transient alteration of large numbers of genes in ZHTc6 cells could reflect the complexity of gene expression regulation by Oct3/4, or may instead reflect the presence of heterogeneous cell populations in ZHTc6 cultures, as evidenced by the upregulation of markers for broader cell types, including primitive/definitive endoderm (*Gata6, Gata4* and *Afp*), mesoderm (*T, Gsc*), and ectoderm (*Cdh2, Mbp, Nefl*) (Supplemental Figure S1, Supplemental Table S4).

To further compare the gene expression changes between the Oct3/4-repression condition (ZHBTc4) and Oct3/4-overexpression condition (ZHTc6), we plotted the fraction of genes in each cluster of ZHTc6 cells present in each cluster of ZHBTc4 cells (Figure 1D). Four major peaks were observed. In ZHBTc4 cells nearly half the genes (69 genes out of 144 genes) in cluster 1 and 20% of the genes (143 out of 667 genes) in cluster 4 were grouped in cluster 10 in ZHTc6 cells (Figure 1D, green arrow). This indicates that genes downregulated under Oct3/4-repression conditions tend to be also downregulated under Oct3/4-overexpression conditions. This group of 212 genes (129 unique gene symbols) included known direct targets of Oct3/4 such as *Spp1/Opn, Sox2, Fbxo15, Otx2*, and *Zfp42/Rex1*, as well as newly identified direct targets of Oct3/4 by genome-wide chromatin immunoprecipitation (ChIP) assays [33], [34] (Figure 1E; Supplemental Table S2). In ZHBTc4 cells, nearly half the genes (84 genes out of 223 genes) in cluster 5 and 13% of the genes (122 out of 906 genes) in cluster 2 were grouped in cluster 1 in ZHTc6 cells (Figure 1D, red arrow). Thus, genes upregulated under the Oct3/4-repression conditions tend to be also upregulated under the Oct3/4-overexpression conditions. This group of 206 genes (180 unique gene symbols) included *Igf2, Krt1-18*, and *Krt2-8*, which are known for their high expression in differentiated cells (Figure 1E; Supplemental Table S3).

The diagram in Figure 1E summarizes this unique mode of gene expression regulation by Oct3/4. Some of the direct Oct3/4 target genes seem to be regulated by Oct3/4 in a bell-shaped dose-response manner or in an inverse bell-shaped dose-response manner. It is thus inappropriate to simply classify all Oct3/4-downstream genes as positively or negatively regulated by Oct3/4. Bell-shaped expression regulation by Oct3/4 has been first demonstrated using tandem Octamer motif promoter constructs [36]. It has also been demonstrated that both low and high levels of Oct3/4 repress the Zfp42/Rex1 promoter, whereas intermediate levels of Oct3/4 expression activate the Zfp42 promoter [24]; Lower panel in Figure 1E). The current results have not only confirmed these earlier works, but established that this is rather common mode of expression regulation by Oct3/4, because many other genes show similar expression patterns. Furthermore, the current analysis has revealed that genes that show the inverse bell-shaped expression pattern exist. That is, low and high levels of Oct3/4 activate the promoter, but intermediate levels of Oct3/4 repress the gene promoter (upper panel in Figure 1E). These gene regulation relationships perhaps underlie a unique mechanism of Oct3/4 regulation, which is required to be maintained in a narrow range of appropriate expression for maintenance of pluripotency: either the increase or decrease of Oct3/4 levels from the intermediate range leads to the differentiation of ES cells [19].

### Identifying Oct3/4 downstream genes

To obtain a list of Oct3/4-downstream candidate genes, we first used Principal Component Analysis (PCA). PCA can dissect multi-dimensional data into a small number of major trends [37]. We have focused here on the more tractable and uniformly-trending microarray results for ZHBTc4 cells, although we have continued the analyses of gene expression in both ZHBTc4 and ZHTc6 and the results of parallel analysis of ZHTc6 cells are presented in Supplementary materials (Supplemental Figure S1, Supplemental Table S1). We concentrated on 3,079 genes that showed statistically significant changes with time by ANOVA statistics (FDR<0.05). Of these, 2,757 non-redundant genes were used for PCA (Supplemental Table S4).

**Figure 2:**
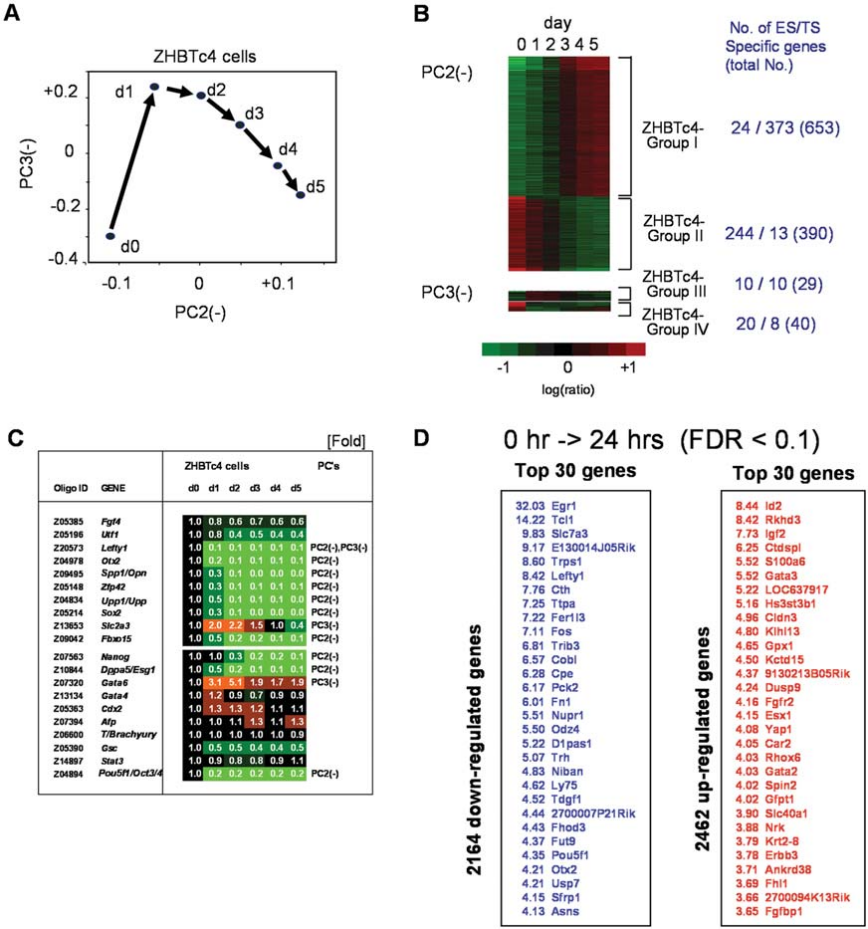
Principal component analysis (PCA) and the expression pattern heatmap of known target genes for Oct3/4 and related genes. (A) 2D-views of PCA for 2,757 genes that were identified as significantly differentially expressed during this time course. (B) The expression pattern and ES/TS specificity of each component of PCA was classified into 4 groups (Group I ∼ Group IV). (C) Known target genes for Oct3/4 and related genes. (D) Top 30 up- and down-regulated genes from 0 h v 24 h.

Figure 2A shows a 2D-plot of results for ZHBTc4 cells over 5 days in PC2(−) and PC3(−) coordinates. The expression patterns of individual genes contributing to each PC are shown in Figure 2B. PC2(−), a major component with *Oct3/4* (an exogenous copy), *Nanog*, and *Krt2-8* genes, represented unidirectional changes (653 genes showing a gradual increase and 390 with a gradual decrease). In contrast, PC3(−), a minor component with *Dnmt3b* and *Gata6* genes, represented transient changes followed by recovery of gene expression (Figure 2B). Genes contributing to PC2(−) seemed to be strongly correlated with the differentiation of ES cells into the trophoblast cell lineage. Our previous microarray analysis comparing the expression patterns of ES cells and Trophoblast Stem (TS) cells [38] identified 1,411 genes as expressed predominantly in ES cells and 1,882 genes as expressed predominantly in TS cells (FDR<0.01) [29], [39]. As expected, a large fraction of genes (373 out of 653) in ZHBTc4-Group I were more highly expressed in TS cells than in ES cells (Figure 2B), whereas a majority of genes (244 out of 390) in ZHBTc4-Group II were more highly expressed in ES cells than in TS cells (Figure 2B, Supplemental Table S4).

Interestingly, most of the genes known to be downstream of Oct3/4, such as *Lefty1, Otx2, Spp1/Opn, Zfp42, Upp1/Upp*, and *Fbxo15*, as well as genes important for pluripotency, such as *Nanog* and *Sox2* (Figure 2C), were included in ZHBTc4-Group II (Figure 2B). These genes were expressed in undifferentiated ES cells and were progressively downregulated as the level of Oct3/4 was repressed (Figure 2B). These results suggests that genes in ZHBTc4-Group II (390 genes) are reasonable candidates for genes downstream of Oct3/4 (Figure 2C). On the other hand, considering the ability to Oct3/4 to activate or repress downstream genes, genes in ZHBTc4-Group I (653 genes) should also be considered to be candidates for genes downstream of Oct3/4 (Figure 2B). These 1043 formed our “list 1” of Oct3/4-downstream candidate genes (Supplemental Table S4).

As an alternative way to identify a list of Oct3/4-downstream candidate genes, we looked for genes with an early response to the repression of Oct3/4 by comparing the gene expression patterns between ZHBTc4 ES cells at 0 hr and 24 hrs of Tet-induction. A stringent statistical criterion (FDR<0.1) identified 2164 down-regulated genes and 2464 up-regulated genes (Supplemental Table S5). These 4628 genes formed our “list 2” of Oct3/4-downstream candidate genes. Thirty genes that showed the highest-fold expression changes are shown in Figure 2D.

### Separating primary target genes from secondary target genes

It is reasonable to assume that the Oct3/4-downstream candidate genes identified here include both primary target genes and the secondary/tertiary target genes, which are regulated by the primary target genes. To delineate Oct3/4-regulated gene cascades or networks, it is important to distinguish primary target genes from those which are indirectly regulated.

One approach is to analyze genomic sequence to search for Oct3/4-binding motifs. However, it is generally difficult to identify primary targets of Oct3/4 by searching for canonical octamer motifs, 5′-ATTA/TGCAT-3′, because it has been shown that Oct3/4 can bind to other similar sequence motifs such as 5′-ACTAGCAT-3′ and 5′-ATCAGCAT-3′ [34], [40]. Furthermore, binding at the octamer motif is not unique to Oct3/4, but common to all Oct gene family members, e.g., Oct1. To reduce the large number of targets identified by the use of degenerate consensus sequence motifs, we searched for clusters of multiple OCT binding sites within −8 kb to +2 kb from transcription start sites of all possible genes in the mouse genome. However, even the search for genes carrying clusters of at least three sites with sequence conservation (>50% compared to the human genome sequence) identified 9326 target genes (Supplemental Table S6), and the search for genes carrying the clusters of at least five sites with the sequence conservation (>50%) and mismatch score (<0.05) identified 4396 target genes (Supplemental Table S7). Without experimental validations of individual Oct3/4-target genes, the utility of genes identified by searching octamer-binding motifs are limited at this point.

**Figure 3:**
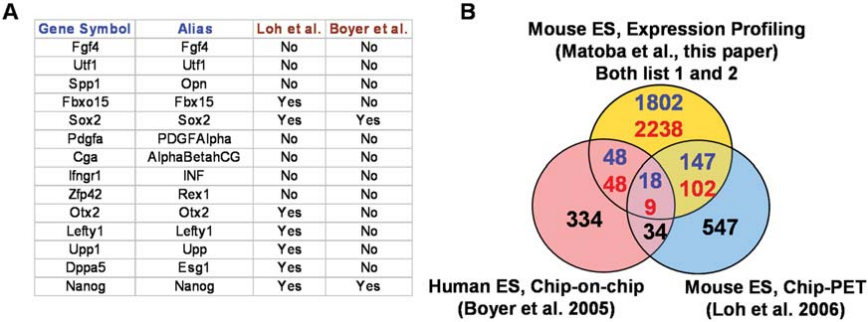
Delineation of Oct3/4-downstream target gene network. (A) A list of genes that have been experimentally demonstrated as the direct downstream targets of Oct3/4. Yes: genes that were also detected by the global ChIP assays. No: genes that were not detected by the global ChIP assays. Mouse homologues of human genes identified in Boyer *et al*. were generated using NCBI HomoloGene Build 49. (B) Comparison of gene lists obtained by the expression profiling (list 1 plus list 2) and global ChIP assays.

**Figure 4:**
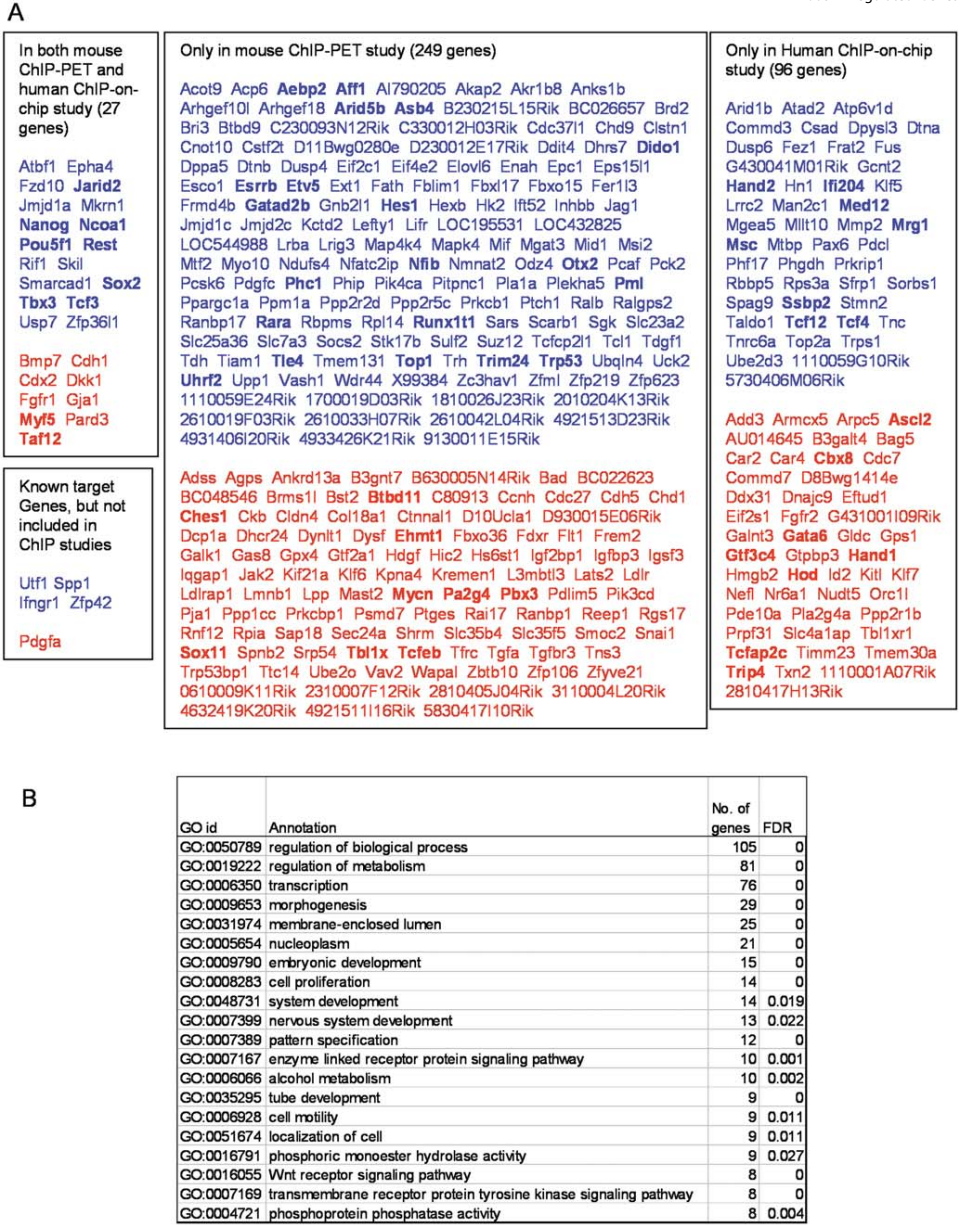
(A) Primary targets of Oct3/4. Genes are separated into four groups: Genes detected by the current expression profiling, mouse ChIP-PET assay, and human ChIP-on-chip assay; previously known target genes that were also detected by the current expression profiling; Genes detected by the current expression profiling and mouse ChIP-PET assay; Genes detected by the current expression profiling and human ChIP-on-chip assay. Genes classified as transcription factors by GO annotation were shown in bold. Genes that were down-regulated by the repression of Oct3/4 in ZHBTc4 cells were shown in blue. Genes that were up-regulated by the repression of Oct3/4 in ZHBTc4 cells were shown in red. (B) Representative GO categories that are enriched in the primary targets of Oct3/4 in a statistically significant manner (FDR>0.05).

Another approach is to use a genome-wide chromatin immunoprecipitation (ChIP) assay and to identify all promoter sequences that are bound by Oct3/4. Recently, Boyer *et al*. have conducted a large scale analysis of promoter binding for OCT4 in human ES cells using ChIP-Chip technologies [33]. They demonstrated that OCT4 was associated with 581 of the promoter regions for known protein-coding genes in the −8 kb to +2 kb region, relative to the transcription start site. Similarly, Loh *et al*. have identified 963 genes as a direct downstream target of Oct3/4 in mouse ES cells using a ChIP-PET (paired-end ditag) assay [34]. We compared our Oct3/4 downstream gene lists with the data by Loh *et al* and Boyer *et al* (Figure 3A). Only a small fraction of genes (61 genes) overlap between human ES [33] and mouse ES cell data [34] (Figure 3A). This is partially due to the biological differences between mouse and human systems as discussed previously [34]. However, some of this effect may also be due to differences in sensitivity and specificity between the techniques used in these studies. In fact, genes that have been experimentally shown to be direct downstream targets of Oct3/4, such as Zfp42/Rex1 [24], were not detected in either Boyer *et al*. or Loh *et al*. (see Figure 3A for a complete list). Thus, we have decided to include all genes that have been shown by either Boyer *et al* or Loh *et al* to be primary targets of Oct3/4 in our list of Oct3/4 target candidates. We compared our combined list (list 1 and list2) with the gene list by Boyer *et al*. and that of Loh *et al*. We found that 372 genes were overlapping with ChIP-based assay results and considered them primary Oct3/4-target genes (Figure 3B). Figure 4A shows gene symbols of 377 primary target genes (372 genes plus five well-known Oct3/4-target genes that were not included in the ChIP-based assays; see Supplemental Table S8 for the details about these genes). The remaining 4040 genes, which were included in either list 1 or list 2 of our Oct3/4-downstream candidate genes, but not included in Oct3/4-binding target by either ChIP-on-chip or ChIP-PET studies, should be considered as the secondary or tertiary targets of Oct3/4. These genes are most likely regulated by the primary target genes of Oct3/4.

Unlike the informatic searching of Oct3/4-binding motifs or ChIP-based assays, expression profiling data can also provide the information about the direction of regulation, i.e., whether the downstream genes are up-regulated or down-regulated by the repression of Oct3/4 in ZHBTc4 ES cells. Out of 396 primary target genes, 159 genes belong to the former category, whereas 213 genes belong to the latter category (Figure 3B, 4A). Out of 4040 secondary/tertiary target genes, 2238 genes belong to the former category, whereas 1802 genes belong to the latter category (Figure 3B). Therefore, the expression profiling of Oct3/4-manipulated ES cells provides important insights into not only the structure, but also the dynamics of Oct3/4-regulated gene networks. The results clearly demonstrate that both ChIP-based assays and expression profiling of Oct3/4-manipulated ES cells are required to delineate the downstream cascades of Oct3/4-regulated genes.

To gain insights into the functions of the 372 primary Oct3/4-downstream candidate genes, we analyzed GO annotations of these genes. Out of these genes, 287 genes had GO annotations representing 160 GO categories with a statistical significance threshold of FDR>0.05 (Supplemental Table S9). Figure 4B shows representative GO categories. Interestingly, one-fourth of the 287 genes were involved in “transcription.” This indicates that Oct3/4 can regulate a large number of genes by regulating many transcription factors, which in turn regulate their downstream genes. Other notable GO categories include “cell proliferation,” which indicates the direct involvement of Oct3/4 in the regulation of cell proliferation. A significant fraction of genes annotated as “enzyme-linked receptor protein signaling pathway” and “Wnt receptor signaling pathway” indicate that Oct3/4 directly regulates key cellular signaling pathways in ES cells. Oct3/4 also directly regulates genes involved in various “system development” sub-categories, such as “nervous system development,” “angiogenesis,” and “lung development.” The direct regulation of the variety of cellular and developmental processes by Oct3/4 is consistent with the profound and diverse effects of Oct3/4 expression levels in ES cells.

### Validation of candidate genes for downstream targets of Oct3/4

Global gene expression analyses of Oct3/4-manipulated ES cells have provided an independent validation to the primary target genes identified *de novo* by the ChIP-on-chip [33] or ChIP- PET [34] analysis, so it stands to reason that the list of genes identified by combining all three data sets, shown in Figure 3B and 4A, is the most reliable at this point. However, to meet more rigorous molecular biology standards, whether these genes are indeed directly regulated by Oct3/4 have to be validated individually by standard molecular biological techniques such as electrophoretic mobility shift assays (EMSA) and luciferase reporter assays. Such validations, however, are not straightforward. It is well-known that genes can be regulated by cis-regulatory elements that are located far from the transcription start sites. Isolating and assaying a few kb upstream region of these target genes would not suffice to conclude whether the genes can be directly regulated by Oct3/4 or not. Especially, it will be difficult to establish that genes cannot be regulated by Oct3/4, because the cis-regulatory elements may not be included in the genomic region assayed *in vitro*. Nevertheless, we tested this strategy to validate at least a gene from this target gene list.

**Figure 5:**
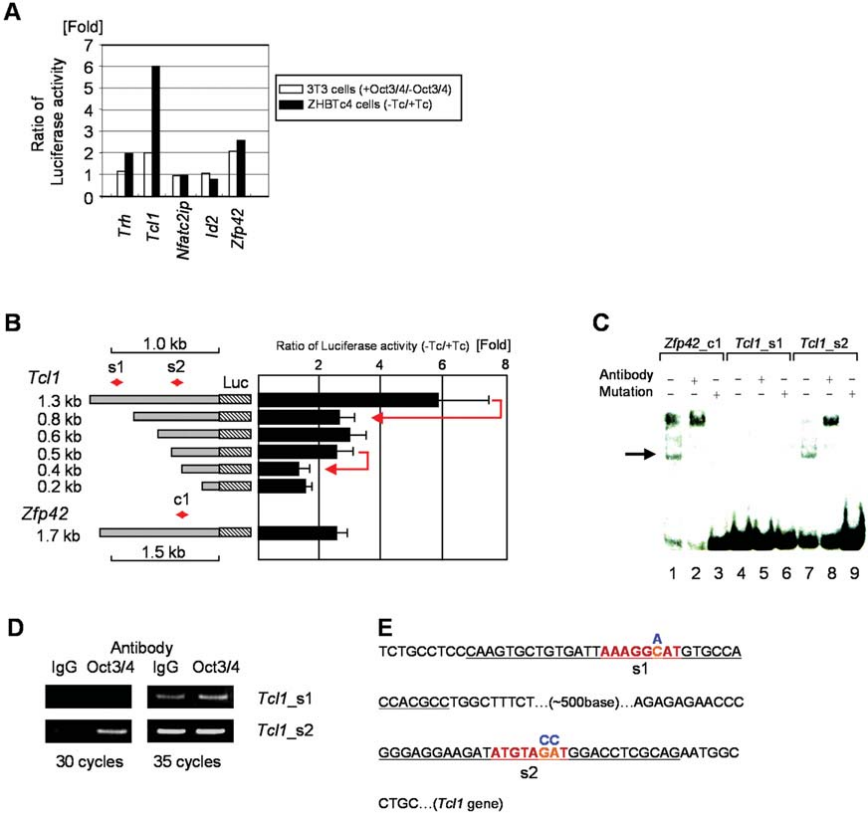
***Tcl1*** **is regulated by Oct3/4**. (A) Luciferase assay for 4 candidate Oct3/4 target genes and one known target gene (*Zfp42*). (B) Luciferase assay for deletion analysis of region upstream of Tcl1 gene. s1 and s2 are candidates for Oct3/4 binding sites, with sequence shown in (E). (C) EMSA for two candidate sites. The arrow indicates the band for Oct3/4/oligonucleotide binding. (D) ChIP assay of two target sites (see Experimental Procedures). (E) The sequence around s1 and s2 sites and the position of mutations for EMSA oligos (blue nucleotides).

First, we selected four putative primary target genes in a rather arbitrarily manner: *Trh, Tcl1*, and *Id2* were selected from top 30 gene lists in Figure 2D and *Nfatc2ip* was selected from a gene list in Figure 4A. We also selected *Zfp42* as a positive control. We amplified by PCR the ∼2 kb upstream putative promoter regions of these genes (Supplemental Figure S2). To investigate whether Oct3/4 can indeed regulate the expression of these genes, we constructed a luciferase reporter gene driven by the upstream putative promoter region and cotransfected it with or without an Oct3/4-expressing plasmid into 3T3 mouse fibroblast cells. Of 5 reporter constructs, the two constructs containing *Tcl1* and *Zfp42* promoters were activated by Oct3/4, as judged by more than 2-fold difference in luciferase activity (Figure 5A). To confirm regulation in a more natural context, we transfected each reporter construct into ZHBTc4 cells, where Oct3/4 levels can be repressed by Tet. Constructs with *Trh, Tcl1*, and *Zfp42* promoters responded to Oct3/4, as judged by more than 2-fold difference of luciferase activity in the presence or absence of Tet (Figure 5A). Notably, *Tcl1* showed even a stronger response than *Zfp42*, a well-known downstream target of Oct3/4 (Figure 5A). These results identified at least *Tcl1*, along with *Zfp42*, as regulated by Oct3/4.

In order to identify Oct3/4 regulatory elements, a series of 5′-deletion mutants of the *Tcl1* promoter were generated and transfected into the ZHBTc4 cells. The luciferase reporter assay revealed that deletion of two regions that cover −0.8 kb ∼ −1.3 kb and −0.4 kb ∼ −0.5 kb, where variant Octamer-binding motifs were found, sharply reduced the responsiveness to Oct3/4 (Figure 5B). We further examined whether Oct3/4 binds to these motifs by electrophoretic mobility shift assays (EMSA) (Figure 5C). As a control, a c1-oligonucleotide that contains the established Oct3/4-binding motif of *Zfp42* was used. For the Oct3/4-binding motifs of *Tcl1*, we used an s1 oligonucleotide that contains an AAAGGCAT motif and an s2 oligonucleotide that contains an ATGTAGAT motif (Figure 5E). The s2 oligonucleotide, but not the s1 oligonucleotide, showed a distinctive band after incubation with ES cell lysates. The band was shifted by Oct3/4 antibody and disappeared when a 2-bp mutation was introduced into the Oct3/4-binding motifs (Figure 5C). Thus, Oct3/4 present in ES cell lysates binds to the s2 oligonucleotide. To further narrow the region, a series of single-base mutant oligonucleotides were generated and subjected to EMSA. The results clearly showed that “ATGTAGAT,” located 410 bp upstream of the *Tcl1* gene, is a functional Oct3/4 binding site (Supplemental Figure S3B, C). To further confirm the binding of Oct3/4 *in vivo*, we performed chromatin immunoprecipitation (ChIP) analysis and demonstrated that Oct3/4 indeed binds to the s2 region, but not to the s1 region (Figure 5D). A luciferase assay using another series of finer deletion mutants of the *Tcl1* promoter further confirmed that the region s2 was indeed an Oct3/4-responsive element in ZHBTc4 cells (Supplemental Figure S3A). Taken together, we conclude that Oct3/4 binds to this variant Oct3/4-binding site (ATGTAGAT) in the *Tcl1* promoter region and directly activates its expression. Interestingly, unlike other Oct3/4-binding sites, there is no Sox2-binding site around this Oct3/4-binding site.

### Functional analysis of Tcl1 in ES cells

**Figure 6:**
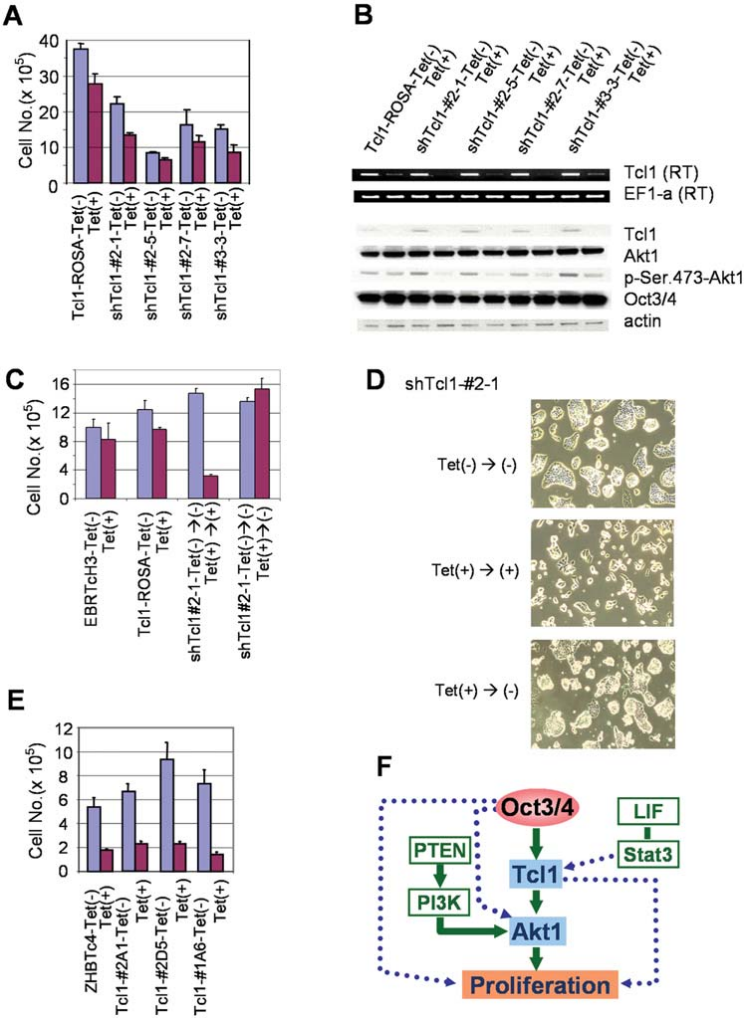
**Functional analysis of Tcl1 in ES cells**. (A) Expression level of Tcl1 gene effects on cell proliferation. The number of ES cells decreased when Tcl1 was knocked down by shRNA. (B) RT-PCR and Western blot analysis of ES cells with shRNA of Tcl1 gene. Tcl1 gene expression affected active Akt1 (p-Ser.473 Akt1), but not wild type of Akt1. (C) Reversibility of Tcl1 downregulation effect. The number of cells was rescued in the absence of Tet. (D) Photomicrographs of ES cell cultures. The number of ES cells decreased when Tcl1 was knocked down by shRNA. Cell proliferation was rescued in the absence of Tet. (E) Overexpression of Tcl1 does not rescue cell proliferation while Oct3/4 is repressed. (F) A model for the involvement of Oct3/4-Tcl1-Akt in ES cell proliferation.

To gain insights into the functional involvement of *Tcl1* in the Oct3/4 pathway in ES cells, *Tcl1* expression was knocked down by transfecting siRNA against *Tcl1* (si*Tcl1*). The proliferation of ES cells was significantly reduced in a dose-dependent manner (Supplemental Figure S4A). Unlike the suppression of proliferation when Oct3/4 expression is repressed in ZHTc4 cells, this suppression of proliferation was not accompanied by any detectable morphological differentiation of ES cells (Supplemental Figure S4A). However, it is conceivable that this rather general proliferation suppression phenotype might be caused by either non-specific or off-target effects of siRNA. To address this issue, we first generated mouse ES cell clones (Tcl1-ROSA) with a Tet-controllable Tcl1 gene by integrating the ORF of *Tcl1* into a ROSA locus [41]. We then established multiple ES cell clones, where a plasmid expressing shRNA against the 3′-UTR of *Tcl1* (shTcl1) was stably transfected into the Tcl1-ROSA ES cell clone. In these mouse ES clones (named shTcl1#2-1, shTcl1#2-5, shTcl1#2-7, and shTcl1#3-3), the expression of endogenous *Tcl1* was constitutively repressed by shRNA, but the expression of exogenous *Tcl1* was repressed only when Tet was added to the cell culture medium. Consistent with the transient siRNA results, the repression of *Tcl1* suppressed the proliferation of ES cells (Figure 6A). The reductions of *Tcl1* in these ES cells at RNA level and protein level were confirmed by the RT-PCR and Western blot (Figure 6B). Because the proliferation of a parental cell line (EBRTcH3) was not suppressed by Tet (Figure 6C), the suppression of proliferation observed here was indeed caused by the repression of *Tcl1*. The suppression of proliferation by the repression of *Tcl1* was also reversible even after 4 days of culture under Tcl1-reduced conditions (Figure 6C, D). This is in sharp contrast to the irreversible effects of Oct3/4-repressed conditions, which not only reduce cellular proliferation, but also differentiate ES cells into the trophoblast lineage.

Further support for the notion that Tcl1 acts on cell proliferation but is not directly involved in the differentiation of ES cells came from the microarray analysis of siTcl1-transfected ES cells. Hierarchical cluster analysis of the selected 1,043 genes that represent PC2(−) in ZHBTc4 cells (Figure 2B) indicated that ZHBTc4 cells showed changes from an expression pattern like ES cells (d0) to a pattern like TS cells (d2) (Supplement Figure S5A, Supplemental Table S10). Unlike ZHBTc4 cells at a comparable time point (d2), siTcl1-transfected ES cells showed an expression pattern like ES cells (Supplemental Figure S5A). In particular, genes involved in cell proliferation regulation showed similar expression changes in siTcl-transfected ES cells and ZHBTc4 cells (Supplemental Figure S5B), whereas trophoblast-lineage markers were not significantly upregulated in siTcl1-transfected ES cells. The microarray analyses were thus consistent with the phenotypic changes observed in ES cells following repression of Tcl1: slowdown of proliferation, but no differentiation towards the trophoblast lineage. Consequently, Tcl1 seems to fractionate the effects of repression of Oct3/4, which causes both the slowdown of proliferation and the differentiation into trophoblast cells.

To investigate whether Tcl1 is a major mediator of the effects of Oct3/4 on the proliferation of ES cells, we established multiple stable cell clones (Tcl1-#2A1, Tcl1-#2D5, and Tcl1-#1A6) by transfecting a plasmid vector constitutively expressing Tcl1 into ZHBTc4 ES cells. In these cells, the repression of Oct3/4 by Tet does not cause the repression of Tcl1, and therefore, the effect of Oct3/4 repression can be uncoupled with the repression of Tcl1. However, the results clearly showed that the constitutive expression of Tcl1 could not prevent the suppression of ES cell proliferation (Figure 6E).

The only known function of Tcl1 protein is to enhance the kinase activity of Akt1 (thymoma viral proto-oncogene 1) in leukemia cells [42]. Akt1 is a key kinase in the Ras/PI3K/Akt1 signaling pathway [43]. To investigate whether Tcl1 also activates Akt1 in ES cells, Western blot analysis was performed. The total amount of Akt1 protein was unchanged, but the active form of Akt1, which is phosphorylated at the Ser-473 site, was reduced in Tcl1-repressed ES cells (Figure 6B). Similar results were obtained in siTcl-transfected ES cells (Supplemental Figure S4B). The suppression of ES cell proliferation by the repression of Tcl1 was restored by the transfection of a plasmid expressing a constitutively active form of Akt1 (caAkt1), suggesting that Akt1 mediates at least some of the proliferation-reducing action of Tcl1 in ES cells. Finally, it is worth mentioning that Oct3/4 expression levels were not changed when Tcl1 expression was repressed (Figure 6B), suggesting that there is no feedback loop from Tcl1 to Oct3/4.

## Discussion

Oct3/4 plays a central role in the maintenance of undifferentiated ES cells, which are characterized by their pluripotency and self-renewal activity. It has also been shown that Oct3/4 plays critical role during preimplantation embryo development and germ cell development. As a first step to understand the diverse roles of Oct3/4, we have carried out global expression profiling of mouse ES cells, where the expression level of Oct3/4 is manipulated using a Tet-inducible system.

### Differentiation of ES cells by the manipulation of Oct3/4

It has been shown previously, by the detection of a few marker genes using Q-PCR, that repression of Oct3/4 leads to the differentiation of ES cells into the trophoblast lineage, and that overexpression of Oct3/4 leads to primitive endoderm and mesoderm differentiation [19], [44]. Our microarray analysis confirms that repression of Oct3/4 differentiates ES cells into the trophoblast lineage by showing the global similarity of gene expression patterns to those of TS cells. However, the analysis indicates that ZHTc6 cells differentiate into more different cell types than appreciated previously, suggesting that overexpression of Oct3/4 alone does not completely determine the fate of ES cells, but may rather facilitate differentiation by perturbing the maintenance of self-renewal. This notion is consistent with recent findings that the effect of Oct3/4 overexpression depends on context. For example, overexpression of Oct3/4 enhances differentiation towards neurons on PA6 stromal cells [45], whereas it enhances differentiation towards beating cardiac muscles in embryoid bodies and hematopoietic cells on OP9 stromal cells (H.N., data not shown). Furthermore, overexpression of Oct3/4 *in vivo* leads to a large array of developmental defects resulting in embryonic lethality [46]. Genes identified here for their correlation to differentiation (PC3(+) in Supplemental Figure S1C and PC2(−) in Figure 2B) should provide good candidate genes for early markers of various lineages.

### A unique mode of gene expression regulation by Oct3/4

One of the key features of Oct3/4-mediated gene expression regulation in ES cells is that the Oct3/4 level has to be maintained at an appropriate level. Either reduction or overexpression of Oct3/4 triggers the differentiation of ES cells [19]. The current work has revealed that many (at least 418) genes, 30 of which are primary targets, are regulated in a peculiar manner: the same gene is activated or repressed depending on the amount of Oct3/4 (Figure 1E). The presence of these “bell-shaped” and “inverse bell-shaped” gene expression regulation relationships indicates that the maintenance of appropriate Oct3/4 levels is built into the gene regulatory network in mouse ES cells.

Two examples of such regulations by Oct3/4 have been reported. First, Scholer *et al*. have shown that a promoter with tandem repeats of octamer motif, which is transcriptionally active in the presence of ordinary Oct3/4 levels, is repressed by excess Oct3/4 [36]. The authors have proposed a “squelching” mechanism, i.e., a factor bridging between Oct3/4 and the basal transcriptional initiation complex is absorbed by excessive amounts of Oct3/4, making it unavailable for transcription [36]. Second, Ben-Shushan *et al*. has shown that the promoter of *Zfp42/Rex1* is activated by ordinary amounts of Oct3/4, but is repressed by the overexpression of Oct3/4 [24]. The authors have pointed out that this peculiar regulatory pattern has also been observed in the case of *Krüppel* [47] and *ATF-1* [48]. Although it is also possible that bell-shaped or inverse bell-shaped expression systems are caused by feedback regulatory loops, which may involve genes acting further downstream, 49 primary Oct3/4 target genes that fall into this category are most likely regulated in a similar manner as *Zfp42/Rex1*.

Because these 49 primary target genes seem to be tightly linked to the requirement of appropriate Oct3/4 levels for the maintenance of the pluripotent/self-renewing state of ES cells, these genes may play a critical role in the ES cells. Indeed, Nanog and Sox2, which are often referred as critical genes for the maintenance of pluripotency and self-renewal in ES cells, are included in the bell-shaped gene expression category (Figure 1E). Similarly, *Esrrb, Fbxo15, Otx2*, and *Spp1*/*Opn* are also frequently referenced as critical to ES cell functions. Interestingly, Jarid2 (Jumonji, AT rich interactive domain 2) and Jmjd1a - two proteins with Jumonji domains–are included in this list. It has been shown recently that proteins with Jumonji domains are involved in chromatin modification [49], especially demethylation of histones [50]. The involvement of Id2, which is grouped in the inverse bell-shaped gene expression category (Figure 1E), in the maintenance of a pluripotent/self-renewing state in mouse ES cells [6] also seems to have important implications. Genes in this list will therefore be important targets for future studies.

### Genes downstream of Oct3/4

The current work clearly demonstrates that both global ChIP assays and global expression profiling are required to understand the gene regulatory network governed by a transcription factor. Global ChIP assay provides the structure, i.e., connections or wiring of the gene regulatory network, whereas the global expression profiling provides the dynamics, i.e., behavior or kinetics of the gene regulatory network. For example, ChIP-on-chip assays of human ES cells [33] and ChIP-PET assays of mouse ES cells [34] have provided a large list of primary target genes whose regulatory sequences, e.g., enhancers/promoters, can be bound by Oct3/4. However, these global ChIP assays cannot provide information about whether Oct3/4 activates or represses the expression of these downstream genes. Such information can be obtained only by doing the global gene expression profiling of ES cells in which the Oct3/4 level is specifically altered. In this report, the global expression profiling of Oct3/4-repressed mouse ES cells uncovered ∼4500 candidate genes as potential downstream targets of Oct3/4. Although it is not easy to distinguish the primary or secondary target genes by global expression profiling alone, the meta-analysis of the combined data sets of global ChIP assays and global expression profiling were able to dissect the downstream gene regulatory network of Oct3/4 as summarized in Figure 3 and 4 (see a complete list in Supplemental Table S8). In this diagram, primary targets and the secondary/tertiary targets of Oct3/4 are separated and the directions (up or down) of regulations are also shown.

While the earlier version of this manuscript was being peer-reviewed and revised for another journal, microarray analyses of mouse ES cells after the repression of Oct3/4 by transient siRNA [34] and stable shRNA [51] were reported. Ivanova *et al*. reported that the expressions of 1133 genes (1072 with gene symbols) are altered [51], whereas Loh *et al*. reported that the expressions of 4620 genes are altered [34]. Considering recent reports on the broad off-target effects of siRNA/shRNA [52]–[54], it is conceivable that these microarray data might include genes, the expressions of which are altered by the repression of genes other than Oct3/4. In contrast, the current microarray data have been obtained from ES cells, where only the Oct3/4 level is manipulated by Tet, and therefore, genes identified as primary or secondary downstream of Oct3/4 in this work are most likely affected directly or indirectly by Oct3/4.

### The Oct3/4-Tcl1-Akt1 pathway

Among these downstream candidate genes, we were able to establish that *Tcl1* is a new direct target gene of Oct3/4, based on several lines of evidence: promoter activity assays by luciferase reporter in *Oct3/4*-cotransfected fibroblast cells and ES cells; direct binding by *in vitro* EMSA assay; and *in vivo* binding assay by ChIP. We also established the sequence 5′-ATGTACAT-3′ as a new variant form of the Oct3/4 binding site.

Tcl1 has been extensively studied as an oncogene in T cell leukemia [55], but our findings suggest that Tcl1 also plays an important role in early mouse embryos and ES cells. This notion is indeed consistent with earlier reports on the expression patterns of Tcl1: it is highly expressed in unfertilized eggs and gradually downregulated during preimplantation development [37]; it is one of the 88 genes whose average expression levels correlated with the gradual loss of developmental potency during development [56]; and it is also one of the ES-enriched genes informatically found together with Nanog [16]. A gene disruption study also showed that a majority of embryos depleted in maternal Tcl1 are unable to reach the blastocyst stage, although Tcl1^−/−^ embryos surviving to birth appear normal except for reduced numbers of lymphocytes in bone marrow, thymus, and spleen [57], [58]. Very recently the involvement of Tcl1 in the regulation of mouse ES cells has also been shown [51].

In this paper, we have shown that the proliferation rate of ES cells is controlled by Tcl1 in a dose-dependent manner: overexpression of Tcl1 increases the ES cell proliferation rate, whereas the repression of Tcl1 reduces it. We have also shown that the forced repression of Tcl1 primarily affects ES cell proliferation, but not differentiation, based on morphology and microarray-based gene expression patterns (Supplemental Table S10). As one of the genes with a bell-shaped expression pattern relative to Oct3/4 level, both overexpression and repression of Oct3/4 reduces the expression level of Tcl1. These data suggest that the reduction of ES cell proliferation caused by both overexpression and repression of Oct3/4 is mediated by Tcl1–a primary target gene of Oct3/4 (Figure 6F). However, it is also clear that Tcl1 is not the only primary Oct3/4 target gene that controls proliferation in ES cells, because the constitutive expression of Tcl1 cannot prevent the reduction of proliferation by the repression of Oct3/4 (Figure 6E). Furthermore, it is important to point out that Oct3/4 is not the only upstream gene for Tcl1, because we have observed that Tcl1 also responds immediately to the activation of Stat3 (Supplemental Figure S6).

It has been shown that Tcl1 enhances the kinase activity of Akt1 [42]. This was originally demonstrated in T-cells [42], [59]–[61], but we find that the level of p-Ser.473-Akt1 is proportional to the level of Tcl1, and thus, the same mechanism also seems to function in ES cells. The rescue of the siTcl1-induced phenotype by a constitutively active form of Akt1 implies that Tcl1 acts through Akt1 in ES cells. Akt1 is one of the key kinases whose activation enhances cell proliferation and inhibits apoptosis. Akt1 associates with PI(3,4,5)P3 at the cell membrane and is activated by phosphorylation [62]. Tensin homologue PTEN suppresses this PI phosphatidylinositol-3-OH-kinase (PI3K) pathway [63]. Similarly, the recently identified *Eras* gene regulates ES cell proliferation through PI3K [64]. Akt1 in turn phosphorylates a number of proteins, including the pro-apoptotic proteins BAD and pro-caspase-9, GSK3, p21WAF1, MDM2, and the forkhead family of transcription factors. Although Akt1 is known to be involved in the control of either cell proliferation or apoptosis, we observed no apoptosis by Annexin-V assays in si*Tcl1*-transfected ES cells (data not shown). Consistent with our findings, apoptosis was also not evident in cleavage blocked *Tcl1* ^−/−^ embryos [57]. It has also been shown recently that the activation of Akt is sufficient to maintain the undifferentiated state of mouse ES cells [65].

### Conclusions

Understanding the global gene network that governs the pluripotency and self-renewal of ES cells is an important first step towards the experimental manipulation of cellular developmental potency. This is also important to understand the global architecture of gene regulatory networks. We have shown in this paper the analysis of dynamic changes in global gene expression patterns of ES cells, in which a specific transcription factor is manipulated so that it can be directly overexpressed or repressed. The approaches used in this work can be applied to almost any transcription factor as well as other key genes functioning in ES cells.

## Materials and Methods

### Growth and differentiation of ES cells

ZHBTc4, ZHTc6, and EB5 ES cells were cultured without feeder cells in LIF-supplemented medium as described [19], [66]. For differentiation, ZHTc6 cells were cultured in the absence of Tet, which induced the overexpression of Oct3/4. ZHBTc4 cells were cultured in the presence of Tet, repressing Oct3/4 expression. Three independent samples were prepared for each time point.

### Microarrays

Microarray experiments were carried out as described [35], [37] using the NIA Mouse 22K Microarray v1.0 (Development 60-mer Oligo: Manufactured by the Agilent Technologies, #011321) [35]. Briefly, 5 µg total RNA was transcribed into double-stranded T7 RNA polymerase promoter-tagged cDNA, then amplified into single-stranded, Cy3- or Cy5-tagged cRNA by T7 polymerase. Each sample for Oct3/4 overexpression was hybridized against the day 0 ZHTc6 sample for 16 hours at 60°C on the microarrays. Oct3/4 repression samples were hybridized against the day 0 ZHBTc4 sample. After washing, microarrays were scanned with an Agilent DNA Microarray Scanner. Dye-swapped hybridization pairs were done for each sample. For comparisons among TS and ES (R1) cells, siTcl1-transfected ES cells, ZHTc6 and ZHBTc4 cells, each sample was hybridized against a common reference pool of RNA. Total RNAs from TS cells [38] and R1 ES cells [67] were kind gifts from Dr. Janet Rossant. All microarray data are available at http://lgsun.grc.nia.nih.gov/ANOVA/ and are also available at the public microarray database (GEO [Accession number: GSE5936] and ArrayExpress [Accession number: E-MNIA-61]).

### Data handling

Feature Extraction software (Agilent Technologies, Palo Alto, California) was used to process scan images and extract quantitative numerical spot data. Of 21,939 spots, 20,251 spots were identified as within 0.03 error valance in the three replications. Cluster analyses were performed using TIGR MEV software (http://www.tigr.org). The χ^2^_ij_ -value of correlation between each cluster (Figure 1D) was calculated by Chi-square statistic (χ^2^_ij_ = (N*f_ij_−S_ij_)2/(N*S_ij_), here S_ij_ = *Σ*f_i_* *Σ*f_j_). Spots were required to have a signal over background of at least 2.5 log (intensity) on average to be included in further analyses. For the ZHTc6 time course, 16,216 spots met this criterion, as did 15,182 spots for the ZHBTc4 time course. A total of 14,690 spots passed in both of the cell lines. Of those data, statistically significant differentially expressed genes were identified using the False Discovery Rate (FDR) method [68] in the NIA Array Analysis software [69]. The FDR was set to 0.05 or 0.01, which corresponds to the average proportion of false positives = 5% or 1%. PCA analysis was performed using FDR = 5% for both cell lines. For comparisons of ZHBTc4 and ZHTc6 cells, TS and ES cells, and siTcl1-transfected ES cells, each data set was combined through a universal control sample.

### Construction of expression vectors, reporter plasmids, and luciferase reporter assay

The Oct3/4 expression vector, pcDNA-Oct3/4, was constructed by inserting a PCR product from ES cell cDNA into the EcoRI site located downstream of the CMV promoter in pcDNA3.1(+) (Invitrogen, Carlsbad, California). Luciferase reporter plasmids were constructed by inserting upstream regions of each gene (see Supplemental Figure S3) into pGL3-Basic vector (Promega, Madison, Wisconsin). All inserts were amplified by genomic PCR and confirmed by sequencing of both of strands. Each PCR primer is described in Supplemental Figure S3. For the luciferase reporter assay in fibroblast 3T3 cells, 40 ng of reporter plasmid DNA was cotransfected into 2×10^4^ cells with 40 ng of pcDNA-Oct3/4, 10 ng of pRL-TK (Promega, Madison, Wisconsin) and 310 ng of pGL3-Basic (Promega, Madison, Wisconsin) using Lipofectamine plus (Invitrogen, Carlsbad, California). Two days after transfection, luciferase activities were measured by the dual luciferase assay system (Promega, Madison, Wisconsin). Triplicate samples were analyzed. The relative activities were calculated from the ratios of luciferase activities in the presence or absence of pcDNA-Oct3/4. Luciferase assays in ES cells were performed using ZHBTc4 cells. The level of Oct3/4 expression can be manipulated in this cell line by a Tet-inducible system [19]. The relative activities were calculated from ratios of luciferase activity in the presence and absence of Tet after 24 hours.

### EMSA and ChIP

Nuclear extracts from ES cells were isolated by using NE-PER Nuclear Extraction Reagents (Pierce Biotechnology, Rockford, Illinois) in the presence of protease inhibitors. DNA mobility shift assays were performed as previously described [40], using nuclear extracts from ES cells and biotinylated oligonucleotides, then transferring to nylon membrane, followed by detection using a Chemiluminescent Nucleic acid detection module kit (Pierce Biotechnology, Rockford, Illinois). Oligo sequences for EMSA: s1, 5′-CGTGGTGGCACATGCCTTTAATCACAGCAC-3′ and 5′-GTGCTGTGATTAAAGGCATGTGCCACCACG-3′; s2, 5′-CTGCGAGGTCCATCTACATATCTTCCTCCC-3′ and 5′-GGGAGGAAGATATGTAGATGGACCTCGCAG-3′. Oct3/4 monoclonal antibody was ‘C-10’ from Santa Cruz Biotechnology (Santa Cruz, California). Chromatin immunoprecipitation was performed using ChIP-IT™ from Active Motif (Carlsbad, California), per the manufacture's protocol, using anti-mouse immunoglobulin G (IgG) as a control and monoclonal antibody of Oct3/4 (C-10) from Santa Cruz Biotechnology (Santa Cruz, California). Fragments of the target site were amplified by PCR with the following primers: 5′-GAATACTGAAGGCCAAGGTC and 5′-AAAGAAAGCCAGGCGTGGTG for s1 site; and 5′-AGAGAGAACCCGGGAGGAAGATA and 5′-CTTATGGTGAGACCCCTAG for s2 site. ExTaq polymerase (Takara, Tokyo, Japan) was used with 30 and 35 cycles in standard protocols.

### siRNA of Tcl1 gene and Western blot analysis

siRNA were co-transfected into EB5 ES cells (2×10^5^ cells/ml) with 100 ng of pPyCAGIP-IRES-puromycin vector using Lipofectamine 2000 (Invitrogen Carlsbad, California). The siRNA sequences were designed as described previously [70]. siRNA sequences for mouse *Tcl1* and for the control (siGFP) are as follows: 5′-GTCATCAAGAGTAATGAAAAATT-3′ and 5′-CAGCCACAACGTCTATATCATGG-3′. One day after transfection, cells were selected by the addition of Puromycin for 24 hours. For western blot analysis, cells were washed with ice cold PBS, then lysed with RIPA buffer (50 mM Tris-Cl, pH7.5, 150 mM NaCl, 0.5% sodium deoxycholate, 1% NP40, 0.1% SDS containing protease inhibitor cocktail (Sigma, St. Louis, Missouri) and phosphatase inhibitor cocktail (Sigma, St. Louis, Missouri)). Whole cell extracts were analyzed on 10% SDS-polyacrylamide gels, followed by immunoblotting on nitrocellulose membranes (Amersham, Piscataway, New Jersey), blocked with 5% non fat dry milk in TBST (Trizma base, 140 mM NaCl, 0.05% Tween-20) and probed with the indicated antibodies at 4°C. All antibodies were purchased from Cell Signaling (Beverly, Massachusetts). For the rescue experiment, EB5 ES cells were transfected with a 1∶9 mixture of each siRNA and either pPyCAG-wtAkt1 (wild type) or pPyCAG-caAkt1 (constitutively active Akt1). The cells were then cultured as described above, and viable cell counts were determined by trypsinizing and staining with trypan blue.

### Isolation of Tcl1 manipulated ES cells

For the isolation of ES cells, which have the overexpression of Tcl1 gene using ROSA-Tet system (Tcl1-ROSA-Tet), we have done as previous described methods [41]. Briefly, 5 µg of circular plasmid DNA of Tcl1 exchange vector (pZhC-Tcl1), which have only cds region of Tcl1 gene, and pCAGGS-Cre were co-transfected into EBRTcH3 ES cells. ES cells were cultured in regular medium within 18 µg/ml zeocin, and then, the 36 clones of ES cells were isolated. Of 36 clones, 12 clones had overexpression of exogenous Tcl1 gene without Tet in the culture medium. For the knocked down of Tcl1 gene by shRNA, we designed the target oligo DNA (5′- CCGCGTCCTGTCGCTGATTAAATTTCAAGAGATTTAATCAGCGACAGGACGTTTTTTGGAAA -3′) having 3′ non-coding region of Tcl1 gene. It will be only downregulated for endogenous Tcl1 expression. After cloning to pSilencer 2.1-U6 vector (Ambion), 5 µg of plasmid DNA were transfected to Tcl1-ROSA-Tet ES cells (2×105 cells/ml) by using Lipofectamine 2000 (Invitrogen). We isolated 24 clones of resistant of puromycin (80 µg/ml), and analyzed the 4 clones, shTcl1-#2-1, #2-5, #2-7 and #3-3.

For overexpression of Tcl1 gene in ZHBTc4 ES cells, at first, amplified PCR products of Tcl1 gene were cloning to pCAG-IRES-puro vector. After confirming the sequences, 5 µg of pCAG-IRES-puro-Tcl1 plasmid DNA were transfected to ZHBTc4 ES cells (2×10^5^ cells/ml) by using Lipofectamine 2000 (Invitrogen). ES cells were cultured in the medium within 80 µg/ml puromycin, and then, the 24 clones of ES cells were isolated. Of 24 clones, three clones were analyzed (Tcl1-#2A1, 2D5 and #1A6).

Western blot analysis and counting the cell numbers have done by same as previous methods.

## Supporting Information

Figure S1 Principal component analysis (PCA) for ZHTc6 cells (A) 2D-views of PCA for 2,757 genes that were identified as significantly differentially expressed during the time course of 5 days. (B) The expression pattern and ES/TS specificity of each component of PCA was classified into 4 groups (Group I∼Group IV). In the PC2(+) axis, the expression of genes contributing to this component (326 genes in ZHTc6-Group I; 1510 genes in ZHTc6-Group II) showed transient responses at days 1 and 2, and then gradually returned to the original state. That the transient response is a major PC is consistent with the results of k-means clustering analysis (see Figure 1B). In contrast, PC3(+) represented unidirectional changes, which seem to correspond to the differentiation of ES cells. Although this is a relatively minor component (116 genes in ZHTc6-Group III; 89 genes in ZHTc6-Group IV), many of these genes were characterized.
(0.90 MB TIF)

Figure S2. Map of the genome for Oct3/4 target candidate genes. For each gene, primer sequences (FW/RV) were used to amplify a target genomic region.
(0.72 MB TIF)

Figure S3. Promoter analysis of upstream of Tcl1 gene(A) Luciferase assay of deletion mutants in ZHBTc4 cells. White box are deletion region. (B) Sequences for promoter analysis. Blue color sequence was used for EMSA (C) EMSA of point mutation (red color in the sequences) for Oct3/4 binding region.
(1.47 MB TIF)

Figure S4. siRNA analysis of Tcl1 gene(A) Photomicrographs of ES cell cultures. The number of ES cells decreased when Tcl1 siRNA was added, but luciferase siRNA (control) showed no effect. (B) Western blot analysis of active Akt1s. The antibody to p-Ser.473 Akt1 detected the active form of Akt1. (C) Wild type Akt1 (wtAkt1) could not rescue Tcl1 siRNA-treated cells, but they were rescued by constitutively active Akt1 (caAkt1).
(1.96 MB TIF)

Figure S5. Gene expression analysis of ZHBTc4, siTcl1, ES and TS cells (A) Hierarchical clustering analysis of 1043 genes that were identified as Group I and II in ZHBTc4 cells. (B) The expression pattern of the genes that were related to cell cycle and trophoblast lineage. siTcl1/siGFP data was shown at day 2 after transfection (see Experimental Procedures).
(1.49 MB TIF)

Figure S6. The effects of LIF on the gene expressions of Tcl1, Trh, Nanog and Socs3. ZHBTc4 ES cells were cultured without feeder cells in LIF-supplemented medium or withdraw LIF. EB5 ES cells were cultured for 24 hours without LIF, and then, added to LIF at time zero. Total RNA was isolated from ES cells by TRIZOL Reagent (Invitrogen). For RT-PCR analyses, cDNA was synthesized from 1 μg of total RNA, with an oligo-dT primer and Moloney murine leukemia virus RT (ReverTra Ace, Toyobo). 1/40 of the single strand cDNA products were used for each PCR amplification with iQ SYBR Green Supermix and iCycler iQ (Bio-Rad) and all data were normalized by expression levels of GAPDH. Primer sets are listed below; Tcl1 S, TTGCTCTTATCGGATGCCATGGCTAC; Tcl1 AS, GGTCTGGGTTATTCATCGTTGGACTC: Trh S, GCGACTCCAAGATGCAGGGACCTTG: Trh AS, CTCTAACCTTACTCCTCCAGAGGTTC: Nanog S, ACCTGAGCTATAAGCAGGTTAAGAC: Nanog AS, GTGCTGAGCCCTTCTGAATCAGAC: GAPDH S, ACCACAGTCCATGCCATCAC: GAPDH AS, TCCACCACCCTGTTGCTGTA.
(0.70 MB TIF)

Table S1. The expression pattern of the 20251 genes in the time course of ZHTc6 and ZHBTc4 cells. Each value was normalized as '0' in time zero.
(5.75 MB XLS)

Table S2. A complete list of genes that show "bell-shaped" expression patterns.
(0.11 MB XLS)

Table S3. A complete list of genes that show "inverse bell-shaped" expression patterns.
(0.10 MB XLS)

Table S4. The 2,757 genes that were used in PCA. The expression values were normalized by median of all log intensities.
(1.14 MB XLS)

Table S5. The expression pattern of the 12198 genes in siRNA of Tcl1 gene (siTcl1). Genes involved in cell proliferation regulation showed similar expression changes in siTcl-transfected ES cells and ZHBTc4 cells. For example, CyclinD1 and Cdk2 were downregulated in both systems, whereas Cdkn1a/P21Cip1 and Rbl2/P130 were upregulated. These expression patterns indicate the suppression of cell proliferation [71]. In contrast, trophoblast-lineage markers were not significantly upregulated in siTcl1-transfected ES cells. For example, Plac1 showed a 2.8-fold increase at day 2 of ZHBTc4 cells, but was not increased in siTcl1-transfected ES cells. Similarly, Krt2-8 showed a 14.3-fold increase at day 2 in ZHBTc4 cells, but very little (1.4-fold) increase in siTcl1-transfected ES cells. The microarray analyses were thus consistent with the phenotypic changes observed in ES cells following repression of Tcl1: slowdown of proliferation, but no differentiation into trophoblast lineage. The Tcl1 thus fractionates the effects of repression of Oct3/4, which causes both the slowdown of proliferation and the differentiation into trophoblast cells.
(2.54 MB XLS)

Table S6 Primary targets of Oct3/4. These were selected based on a search for multiple OCT binding sites within 8000 to +2000 bp. The first file requires at least 3 sites with conservation >50%
(2.19 MB XLS)

Table S7 At least 5 sites with conservation >50%, and mismatch score <0.05.
(1.04 MB XLS)

Table S8 A list of genes containing gene lists from the PCA analysis (Matoba et al., this paper), 0 hr vs 24 hrs analysis (Matoba et al, this paper), human ES ChIP-on-chip (Boyer et al., 2005), mouse ES ChIP-PET (Loh et al, 2006).
(7.13 MB XLS)

Table S9 A list of 160 statistically significant GO categories.
(0.15 MB XLS)

Table S10 Comparison between siTcl1 and ZHTc6/ZHBTc4 cells. The 325 genes, which were up- or down-regulated in siTcl1, were classified into the groups of PCA in ZHTc6 or ZHBTc4 cells.
(1.45 MB XLS)
