# Supplementary figures and images for: Dissecting Oct3/4-Regulated Gene Networks in Embryonic Stem Cells by Expression Profiling
Source: PLoS One. 2006 Dec 20;1(1):e26. doi: 10.1371/journal.pone.0000026 (PMC1762406; doi:10.1371/journal.pone.0000026)

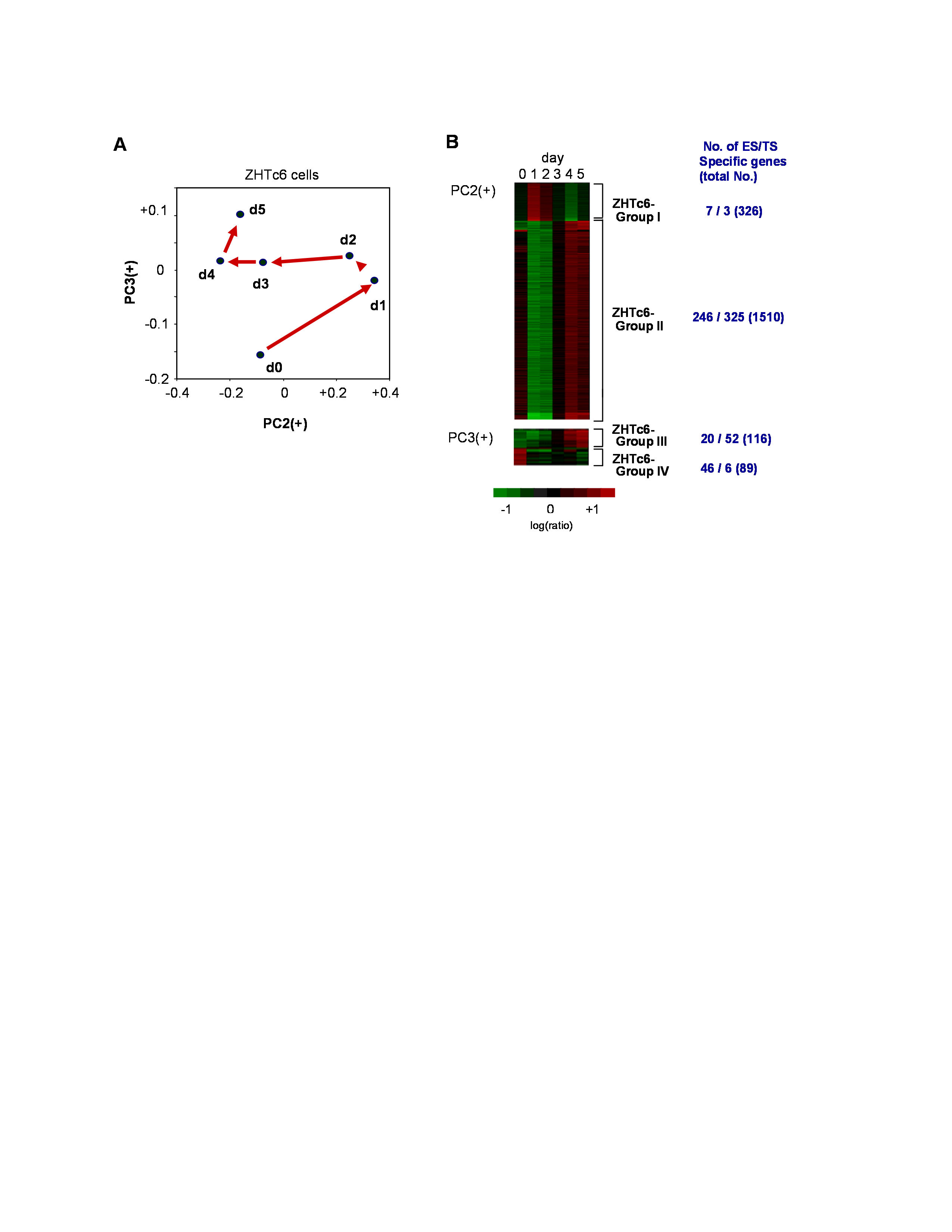

Supplement: Figure S1 — Principal component analysis (PCA) for ZHTc6 cells (A) 2D-views of PCA for 2,757 genes that were identified as significantly differentially expressed during the time course of 5 days. (B) The expression pattern and ES/TS specificity of each component of PCA was classified into 4 groups (Group I∼Group IV). In the PC2(+) axis, the expression of genes contributing to this component (326 genes in ZHTc6-Group I; 1510 genes in ZHTc6-Group II) showed transient responses at days 1 and 2, and then gradually returned to the original state. That the transient response is a major PC is consistent with the results of k-means clustering analysis (see Figure 1B). In contrast, PC3(+) represented unidirectional changes, which seem to correspond to the differentiation of ES cells. Although this is a relatively minor component (116 genes in ZHTc6-Group III; 89 genes in ZHTc6-Group IV), many of these genes were characterized. (0.90 MB TIF) [file pone.0000026.s001.tif]

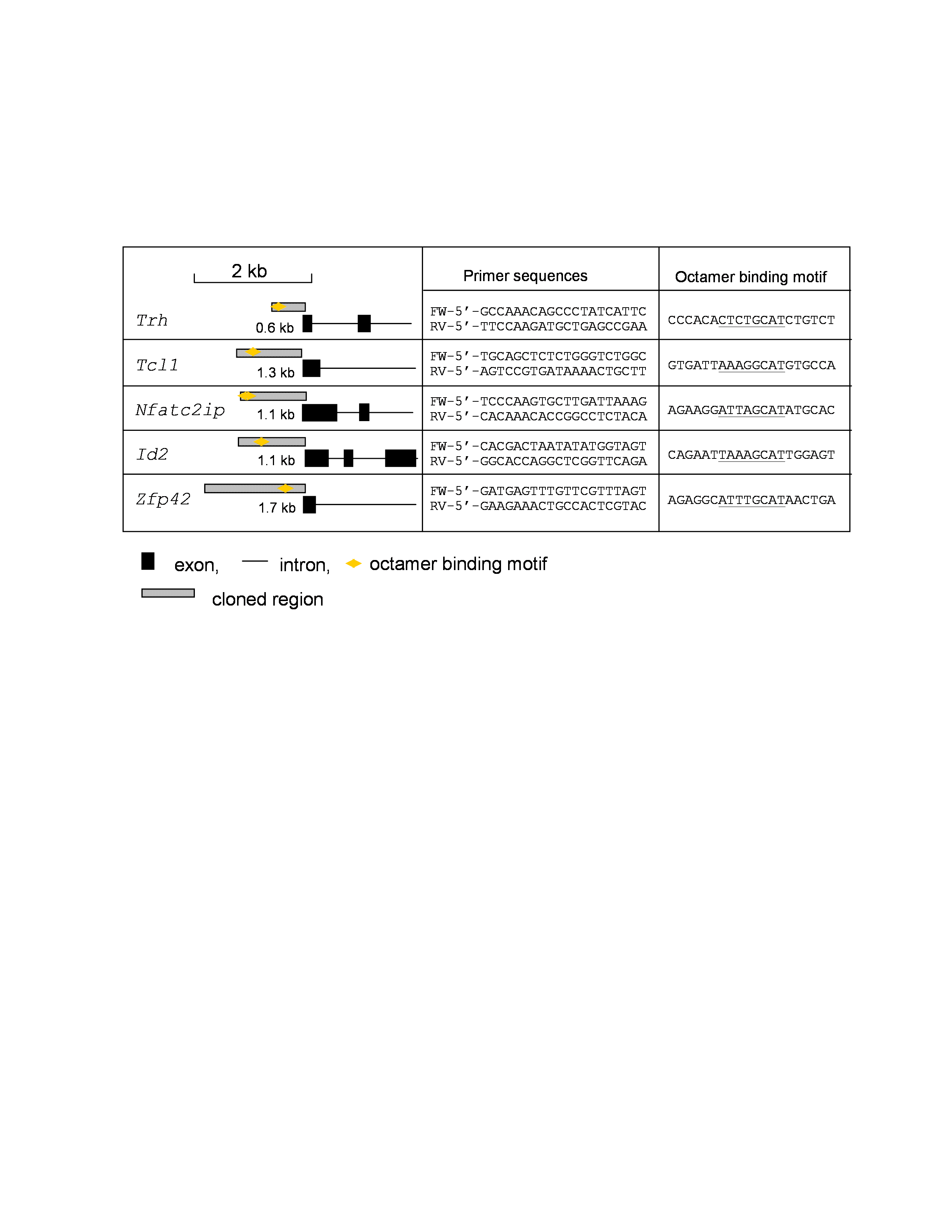

Supplement: Figure S2. — Map of the genome for Oct3/4 target candidate genes. For each gene, primer sequences (FW/RV) were used to amplify a target genomic region. (0.72 MB TIF) [file pone.0000026.s002.tif]

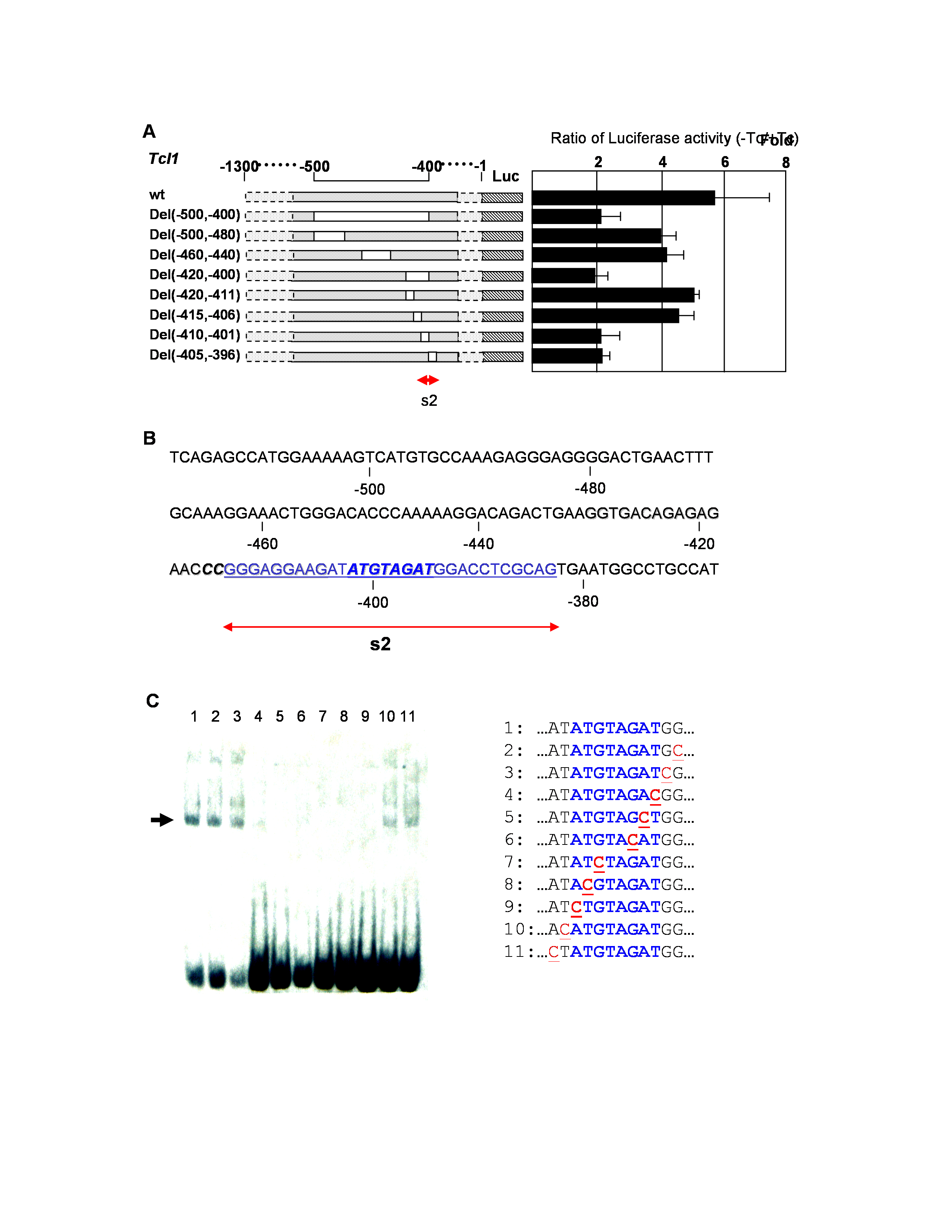

Supplement: Figure S3. — Promoter analysis of upstream of Tcl1 gene(A) Luciferase assay of deletion mutants in ZHBTc4 cells. White box are deletion region. (B) Sequences for promoter analysis. Blue color sequence was used for EMSA (C) EMSA of point mutation (red color in the sequences) for Oct3/4 binding region. (1.47 MB TIF) [file pone.0000026.s003.tif]

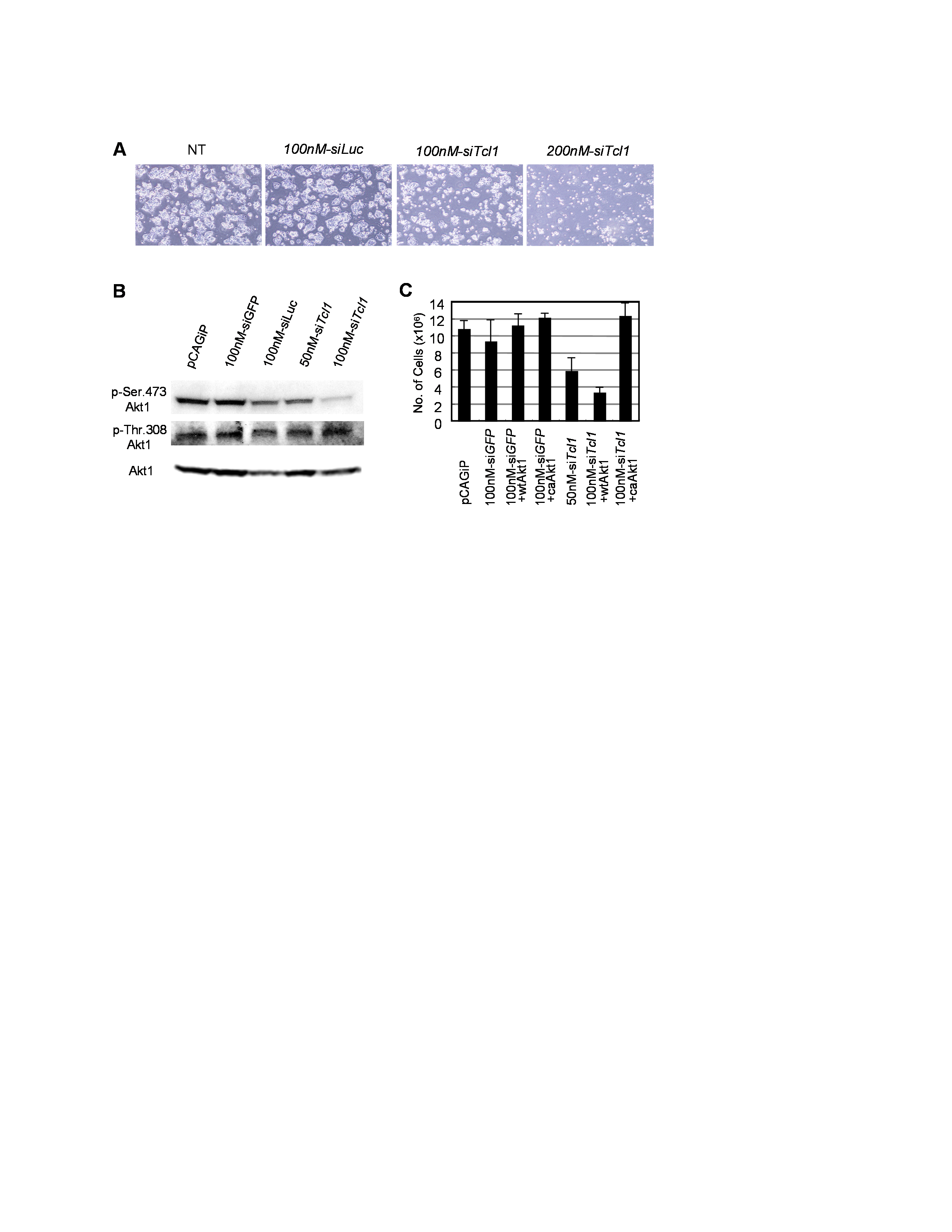

Supplement: Figure S4. — siRNA analysis of Tcl1 gene(A) Photomicrographs of ES cell cultures. The number of ES cells decreased when Tcl1 siRNA was added, but luciferase siRNA (control) showed no effect. (B) Western blot analysis of active Akt1s. The antibody to p-Ser.473 Akt1 detected the active form of Akt1. (C) Wild type Akt1 (wtAkt1) could not rescue Tcl1 siRNA-treated cells, but they were rescued by constitutively active Akt1 (caAkt1). (1.96 MB TIF) [file pone.0000026.s004.tif]

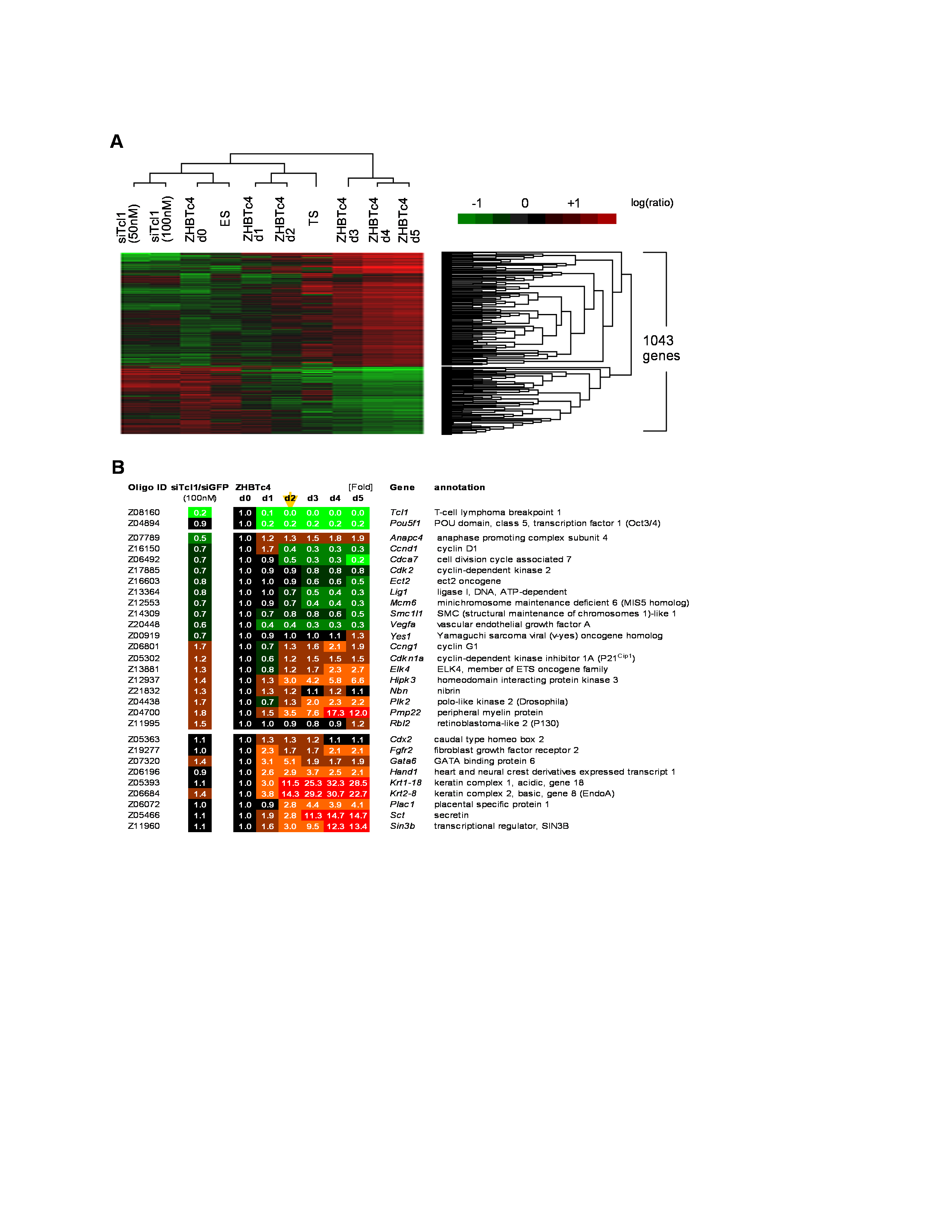

Supplement: Figure S5. — Gene expression analysis of ZHBTc4, siTcl1, ES and TS cells (A) Hierarchical clustering analysis of 1043 genes that were identified as Group I and II in ZHBTc4 cells. (B) The expression pattern of the genes that were related to cell cycle and trophoblast lineage. siTcl1/siGFP data was shown at day 2 after transfection (see Experimental Procedures). (1.49 MB TIF) [file pone.0000026.s005.tif]

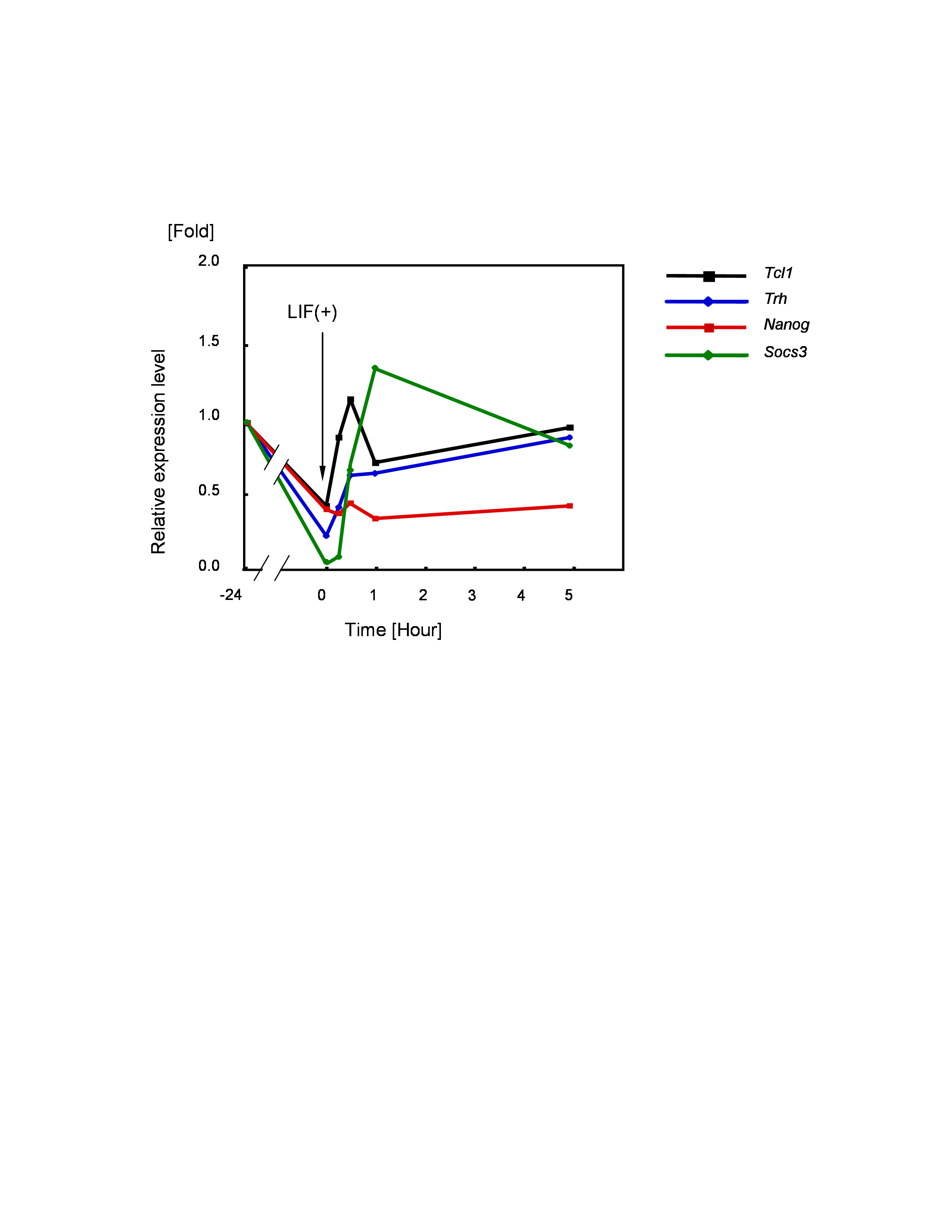

Supplement: Figure S6. — The effects of LIF on the gene expressions of Tcl1, Trh, Nanog and Socs3. ZHBTc4 ES cells were cultured without feeder cells in LIF-supplemented medium or withdraw LIF. EB5 ES cells were cultured for 24 hours without LIF, and then, added to LIF at time zero. Total RNA was isolated from ES cells by TRIZOL Reagent (Invitrogen). For RT-PCR analyses, cDNA was synthesized from 1 μg of total RNA, with an oligo-dT primer and Moloney murine leukemia virus RT (ReverTra Ace, Toyobo). 1/40 of the single strand cDNA products were used for each PCR amplification with iQ SYBR Green Supermix and iCycler iQ (Bio-Rad) and all data were normalized by expression levels of GAPDH. Primer sets are listed below; Tcl1 S, TTGCTCTTATCGGATGCCATGGCTAC; Tcl1 AS, GGTCTGGGTTATTCATCGTTGGACTC: Trh S, GCGACTCCAAGATGCAGGGACCTTG: Trh AS, CTCTAACCTTACTCCTCCAGAGGTTC: Nanog S, ACCTGAGCTATAAGCAGGTTAAGAC: Nanog AS, GTGCTGAGCCCTTCTGAATCAGAC: GAPDH S, ACCACAGTCCATGCCATCAC: GAPDH AS, TCCACCACCCTGTTGCTGTA. (0.70 MB TIF) [file pone.0000026.s006.tif]
